# How Valuable Lives Are Lost

**Published:** 1882-12

**Authors:** 


### HOW VALUABLE LIVES ARE LOST.

Hardly a day passes in this climate of ours, between the chilly days of autumn and the month of June, that the community is not startled by the announcement of the sudden death of some prominent man, who has been at the head, perhaps, of vast enterprises. We feel almost certain, before making the inquiry, that the immediate cause of death was pneumonia. The obituary probably repeats that thread-bare untruth about the "inscrutible mysteries of Providence." There is really no mystery about it. Business men, and particularly Americans, are so intent on pushing their business that they lose sight of all else. They think they have no time to be ill, and in many instances they only realize the danger of delay when the shadow of death is upon them. Had they less ambition to "die rich" they would be more likely to heed the warnings of danger that are always given, and then not one case of pneumonia in one hundred would prove fatal. Any person—and particularly one over forty years of age—who neglects a severe cold at this season of the year is in danger of pneumonia.

The only safe plan is to remain in a warm, comfortable room, or, in severe cases, in bed, employing such remedies as may be prescribed by a competent physician. If there is delay in taking care of one's self until the inflammation is established, there is great danger of a fatal result.

When death, the great reconciler, has come, it is never our tenderness that we repent of, but our severity.—*George Eliot.*

